# Harnessing Phones to Target Pediatric Populations with Socially Complex Needs: Systematic Review

**DOI:** 10.2196/19269

**Published:** 2020-08-26

**Authors:** Colleen Stiles-Shields, Lauren M Potthoff, Dawn T Bounds, Maureen T S Burns, Janel M Draxler, Caitlin H Otwell, Emily D Wolodiger, Jennifer Westrick, Niranjan S Karnik

**Affiliations:** 1 Section of Community Behavioral Health Department of Psychiatry and Behavioral Sciences Rush University Medical Center Chicago, IL United States; 2 Department of Gastroenterology Pritzker Department of Psychiatry and Behavioral Health Ann & Robert H Lurie Children’s Hospital of Chicago Chicago, IL United States; 3 Sue & Bill Gross School of Nursing University of California Irvine Irvine, CA United States; 4 Boston Children's Hospital Boston, IL United States; 5 Autism Assessment, Research, Treatment & Services Center Department of Psychiatry and Behavioral Sciences Rush University Medical Center Chicago, IL United States; 6 Library of Rush University Medical Center Chicago, IL United States

**Keywords:** underserved youth, digital mental health, mHealth, telehealth, health disparities

## Abstract

**Background:**

Mobile and smartphones are owned and accessed by many, making them a potentially optimal delivery mechanism to reach pediatric patients with socially complex needs (ie, pediatric populations who face overlapping adversities).

**Objective:**

To address the specialized needs of youth from such groups, this review synthesized the literature exploring the use of phone-based delivery to access pediatric populations with socially complex needs, targeting mental and behavioral health outcomes. The purpose of this synthesis was to provide recommendations for future research developing phone-based interventions for youth with socially complex needs.

**Methods:**

A trained medical librarian conducted the search strategy in the following databases: PubMed, Scopus, CINAHL, PsycINFO, Cochrane CENTRAL Register of Controlled Trials, Cochrane Database of Systematic Reviews, and Google Scholar. Studies targeting youth with socially complex needs were defined by recruiting samples that were primarily from traditionally underserved populations (ie, sex/gender minorities, racial/ethnic background, low socioeconomic status, rural/remote location, and sexual orientation). A systematic narrative framework was utilized and the Preferred Reporting Items for Systematic Reviews and Meta-Analyses guidelines were followed (registration number CRD42020141212).

**Results:**

A total of 14 studies met the inclusion criteria, with 3 depicting the use of phones to complete assessment and tracking goals and 11 to intervene on mental and behavioral health targets.

**Conclusions:**

The literature indicates important directions for future research, including (1) involving diverse and representative teens (ie, the likely users of the interventions), stakeholders, and clinical/research staff; (2) integrating evidence-based therapies with minority-focused theories; (3) harnessing mobile device capabilities; and (4) considering and assessing for potential costs in phones as delivery mechanisms.

**Trial Registration:**

PROSPERO CRD42020141212; https://www.crd.york.ac.uk/prospero/display_record.php?RecordID=141212

## Introduction

### Background

Pediatric populations with socially complex needs are likely to have their behavioral health negatively impacted—from having a lower health status than peers to being less likely to receive appropriate physical and mental health diagnoses [1]. The term “socially complex needs” is used to describe pediatric populations that face overlapping adversities [2]. Such youth may endure adverse childhood experiences, physical symptoms (eg, pain), or be from traditionally underserved populations (eg, low socioeconomic status [SES], belonging to a minority racial/ethnic or gender/sexual minority group, or living in isolation from accessible services [3]). The behavioral health disparities associated with having socially complex needs have lasting detrimental effects, including a higher likelihood of chronic illness in adulthood [4]. For these reasons, repeated calls to promote the behavioral health needs of such pediatric populations have been made [5-10].

The ubiquity of mobile and smartphone access has promoted refrains about the promise of digital mental health tools to overcome access barriers to pediatric behavioral health interventions [11]. While some populations are less likely to adopt computer use and are more likely to lack home broadband access [12], nearly all American adults report owning a mobile phone (96%) [13] and 95% of teens report owning or having access to a smartphone [14]. Therefore, pediatric interventions that harness phones as a delivery mechanism (ie, mobile health [mHealth] and telehealth) may be more likely to successfully access pediatric patients with socially complex needs [13]. However, due to issues such as data plan costs, service lapses, and lower phone literacy, a “digital divide” is occurring that could further perpetuate disparities in the use of phones as a delivery mechanism [15-19]. Indeed, if not appropriately evaluating tools with socially complex populations and adapting designs to fit their user and access needs [20], researchers and clinicians are missing the opportunity to reach pediatric populations through a medium that young people are already using for other purposes (eg, using a smartphone to interact with social media).

### Purpose

While more research is critically needed, some work to harness phones as a delivery mechanism specifically for socially complex populations has begun. As the field shifts to adapt such tools to be more inclusive, synthesis of the small but existing literature may be beneficial. Indeed, this synthesis may promote increasing adaptations of such tools for pediatric populations with socially complex needs and avoid potentially superfluous evaluations that would delay deployment to youth in need of support and care. Therefore, to provide recommendations for future research developing inclusive interventions, this study systematically reviewed the literature for multiple criteria. First, a focus on phone-based interventions (mHealth and telehealth) was made. While the field has moved toward a focus on digital health technologies (eg, harnessing mobile or smartphones to deliver monitoring or intervention activities), telehealth interventions that involve calling participants—even on landlines—were included. These criteria were used because interventions using telephone calls to reach youth may still be applicable as smartphones have phone call capabilities. Second, this review focused on mental (eg, mood) and behavioral health targets (eg, physical activity) for pediatric patients with socially complex needs. As any pediatric patient is likely to have socially complex needs (eg, managing symptoms associated with acute or chronic conditions), we operationalized studies that targeted pediatric patients with socially complex needs as those that recruited samples primarily comprising (ie, ≥50%) youth from traditionally underserved populations (eg, low SES, belonging to a minority racial/ethnic or gender/sexual minority group, or living in isolation from accessible services [3]). The synthesis of these findings was used to define (1) uses of phone-based delivery practices; (2) culturally specific tailoring practices; (3) applications of evidence-based skills and grounded theories to inform design; and (4) additional supports that promote comfort, use, or intended intervention outcomes for pediatric populations with socially complex needs.

## Methods

### Search Strategy

The review was conducted and reported following the Preferred Reporting Items for Systematic Reviews and Meta-Analyses (PRISMA) statement and checklist [21] and was registered prior to data extraction in PROSPERO (registration number CRD42020141212). No limitations were put on the search in terms of language, date of publication, or geographic area. The search strategy included controlled vocabulary (ie, MeSH terms) and keywords in the title or abstract fields. Seven electronic databases were searched, including PubMed, Scopus, CINAHL, PsycINFO, Cochrane CENTRAL Register of Controlled Trials, Cochrane Database of Systematic Reviews, and Google Scholar. The search strategy was conducted collaboratively by the lead author (CS-S) and a trained medical librarian (JW) and the literature search was conducted by a trained medical librarian (JW) in August 2019 (Multimedia Appendix 1).

### Inclusion and Exclusion Criteria

For inclusion in the review, studies were required to (1) specifically target a pediatric population with socially complex needs (as noted above, this was operationalized by the majority [>50%] of the sample belonging to an underserved group [eg, minority population, low SES, rural geographical location] [3]); (2) utilize a phone (ie, smartphone, cellphone, landline, SMS text messages, push notifications, gathering passive data, or have a user access an app[s]) as a means of targeting youth (phones could be used as the sole delivery mechanism or as part of a multimethod intervention delivery); (3) report outcomes related to mood (eg, depression, sadness, low mood), anxiety/stress, or wellness (eg, exercise, diet, sleep, treatment adherence); (4) include samples that were at least 50% under the age of 18; and (5) be written in English. Technical validation papers reporting on the development of digital mental health interventions, conference abstracts, review papers, and samples fewer than 20 were excluded.

### Study Selection

Literature search results were uploaded into Covidence, a not-for-profit, online systematic review service partnered with Cochrane [22]. From the initial search results, all titles and abstracts were independently screened by 2 reviewers against the inclusion criteria. Following this, full-text articles were also reviewed by 2 independent reviewers. Any discrepancies about inclusion at either stage were resolved through consensus with a third reviewer.

### Data Extraction

Reviewer teams (CS-S, LP, DB, MB, JD, CO, EW) extracted data (eg, sample characteristics, use of phone, primary outcomes) independently and in duplicate from each eligible study using an online extraction form designed by the lead author (CS-S) using Google Forms. Discrepancies were, again, resolved through consensus.

### Quality Assessment

The Cochrane Collaboration’s tool for assessing risk of bias in randomized trials was used to assess the risk of bias for selection, performance, detection, attrition, and reporting [23]. Specifically, each study that was included in the final review was independently rated in duplicate for each form of bias.

### Data Synthesis

Because of the variability in outcome measures and methodologies, a meta-analytic approach was deemed inappropriate for the current review. Alternatively, a systematic narrative framework was utilized, with results classified under “Tracking and Assessment” or “Intervention.” To best inform the design of future interventions for pediatric populations with socially complex needs delivered through phones, the systematic narrative synthesis included *population-specific tailoring, evidence-based skills and theories* (interventions only), *use of phones for delivery,* and *additional support*. These categories were selected prior to data collection as they would provide key insights from the literature into development decisions made for specific user needs relating to pediatric populations with socially complex needs. Finally, to contextualize the findings, *study characteristics, primary outcomes,* and *usage and acceptability* were also included.

## Results

### Included Studies

**Figure 1:**
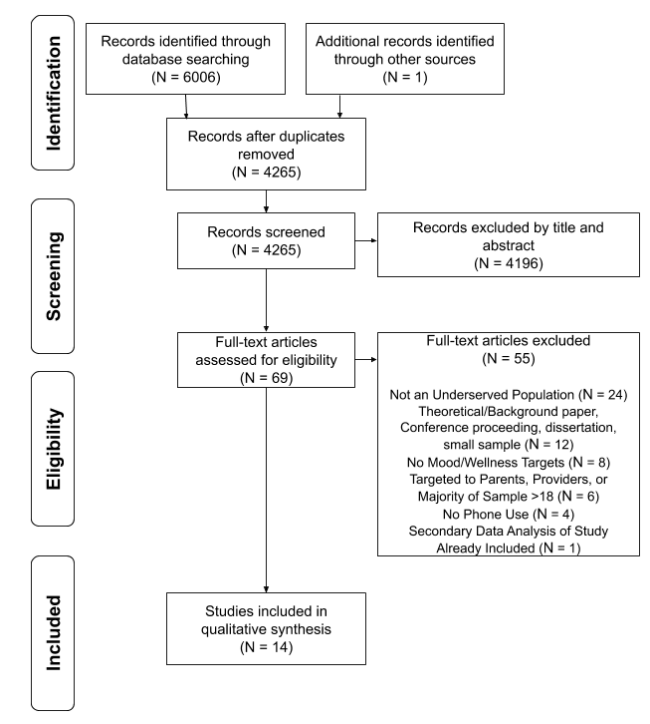
PRISMA flow diagram for study inclusion. aAdapted from “Preferred Reporting Items for Systematic Reviews and Meta-Analyses: The PRISMA Statement,” by D. Moher, A. Liberati, J. Tetzlaff, D. G. Altman, and The PRISMA Group, 2009, PLoS Med. 6(7), e1000097.

Following the removal of duplicates identified by Covidence, 4626 titles and abstracts were independently reviewed in duplicate by 2 reviewers. A total of 69 full-text articles were reviewed in duplicate for inclusion, with 14 articles selected for data extraction. See Figure 1 for the PRISMA flow diagram. Of the 14 studies, 3 focused on tracking and assessment and 11 were intervention based. Findings from the 3 *Tracking and Assessment* studies will be discussed, followed by findings from the 11 *Intervention* studies, and finally, the outcomes of the quality of all included studies will be presented.

**Table 1:** Study characteristics.

Classification and authors (year) [Reference]		Target condition, population/outcome		Name	Modality	Additional support	N (Intervention)	Age, % Female
**Tracking and Assessment**								
	Bakshi et al (2017) [18]	SCD^a^/Pain symptoms management tracking		Not applicable	SMS text message, Web EMA^b^	PRN^c^ (A)^d^	20	12-22, 75
	Jacob et al (2012) [19]	SCD/Pain symptoms management tracking		Wireless Pain Intervention Program	Mobile Web Page	PRN (A)	67	10-17, 54
	Odgers and Russell (2017) [20]	At-risk, low SES^e^/violence exposure + behavioral health		miLife	Mobile EMA	No	151	11-15, 48
**Intervention**								
	DiClemente et al (2014) [21]	African American teen girls/Safe sex behaviors		HORIZONS	Phone call^f^	Yes	701 (342)	14-20, 100
	Leonard et al (2018) [22]	Homeless moms/Emotion regulation		Calm Mom	App^f^	Yes + A	49	13-21, 100
	Nollen et al (2014) [23]	Low SES, minority Girls/Obesity-related behaviors		NR^g^	App^f^	No	51 (26)	9-14, 100
	Perry et al (2017) [24]	Low SES, minority teens/Asthma		NR	App^f^	No (A)	34 (17)	14-16, 38
	Reid et al (2011) [25]	Rural primary care/emotional self-awareness, mood, anxiety		mobiletype	App, EMA^f^	PRN	114 (68)	14-24, 72
	Rokicki and Fink (2017) [26]	At-risk teen girls/Safe sex behavior knowledge		NR	SMS text message^f^	No (A)	498 (205)	14-24, 100
	Schatz et al (2015) [27]	SCD/Pain coping		NR	App, phone call	Yes	46 (23)	8-21, 59
	Seid et al (2011) [28]	Low SES, minority teens/Asthma		NR	SMS text message^f^	Yes + A	26 (14)	12-18, 69
	Smith et al (2014) [29]	Low SES teen boys/Obesity-related behaviors		ATLAS	App^f^	Yes	361 (181)	12-14, 0
	Thompson et al (2016) [30]	Minority teens/Obesity-related behaviors		NR	SMS text message^f^	Yes + A	160 (120)	14-17, 52
	Ybarra et al (2017) [31]	Sexual minority teen boys/Safe Sex behavior, HIV prevention		Guy2Guy	SMS text message^f^	Yes + A	320 (150)	14-18, 0

^a^SCD: sickle cell disease.

^b^EMA: ecological momentary assessment.

^c^PRN: as needed.

^d^A: automated support.

^e^SES: socioeconomic status.

^f^Explicitly detailed use of population-specific tailored messaging or design practices.

^g^NR: not reported.

### Tracking and Assessment

#### Study Characteristics

Three studies targeted mood, anxiety, or wellness outcomes for socially complex pediatric populations through tracking and assessment. Specifically, 2 studies focused on pain tracking for African American youth with sickle cell disease (SCD) [24,25]. The third study focused on violence exposure and psychosocial factors for adolescents from low SES neighborhoods who also presented with at least three parent-reported risk factors (ie, behavioral difficulties, inattention and hyperactivity, early initiation of substance use, or having a parent with a substance misuse issue) [26]. All of the samples were recruited from the United States of America and ranged in size from 20 to 151. Two samples were primarily female [24,25] and 1 was minority female [26]. See Table 1 for study characteristics.

#### Population-Specific Tailoring

Of the 3 studies, only Bakshi et al [24] directly reported or cited prior work discussing the use of tailoring the study to a particular population’s needs. Specifically, cognitive interviewing techniques were used to ensure content validity of the messaging (eg, removing fatigue from assessment queries, as it not associated with experience of patients with SCD), semistructured interviews were conducted for feedback on content and design layout (eg, change the workflow so as to not assess the impact of pain on schoolwork if there were no assignments that day), and user reviews following site creation (eg, demonstrated acceptability) were completed with adolescents and young adults with SCD for a web-based multidimensional pain diary [38]. In addition, participants who might be unable to use/access the web-based platform had the option to transmit pain reports via SMS text message [24].

#### Use of Phones for Delivery

The 3 studies utilized mobile phones or smartphones in some way to deliver their assessment. Bakshi et al [24] employed a web-based ecological momentary assessment (EMA) platform for their pain intensity diary; however, they included SMS text messaging of pain reports to overcome barriers to accessing the webpage. While Jacob et al [25] also utilized a web-based diary system, they provided smartphones with wireless packages to all participants to enter data. Similarly, Odgers and Russell [26] provided smartphones preprogrammed to alert three times within each participant’s schedule.

#### Additional Support

Support beyond the described use of phones was included in the methodology of 2 studies. First, Bakshi et al [24] described having study staff contact participants with SCD if a pain report surpassed a predetermined rating threshold. This support was intended to promote pain management assistance from a provider or hospital [24]. Second, Jacob et al [25] reported having (1) participants with SCD attend an in-person information session on utilizing smartphones to access the e-Diary; (2) an advanced practice registered nurse monitor symptoms and contact participants if reports reached clinical elevations; (3) unlimited SMS text message and phone call support options for participants to contact the advanced practice registered nurse; and (4) technology support as needed.

#### Primary Outcomes

The primary outcome for the studies tracking SCD symptoms was pain. However, the 2 studies reported these findings differently. Bakshi et al [24] reported that their participants with SCD endorsed having pain on the majority of days (76.2%); 30% of participants had all of their entries indicating pain, whereas another 30% primarily denied having pain for most of the time. Jacob et al [25] reported that over half of all diary entries included pain (55%) and that their participants with SCD had a mean pain rating of 4.1 (SD 2.2; range 1-10, with 10 being highest), with no evidence to suggest differences from morning and evening pain, nor by age (10-13 vs 14-17 years). Odgers and Russell [26] identified that 75% of their sample was exposed to violence on at least one day, and reported depressive symptoms about a quarter of the time, anger or irritability nearly 15% of the time, conduct problems about 7% of the time, and health-risk behaviors about 13% of the time. In addition, anger, depression, and conduct problems were more likely to be reported on violence-exposed days and depressive symptoms were more common on days following violence exposure [26]. See Multimedia Appendix 2 for study outcome details.

#### Usage and Acceptability

Usage and acceptability reports were also variable across studies. EMA was completed the majority of time for Bakshi et al [24], with more than 85% of study days associated with 2 or more completed EMAs. During focus groups, participants reported positive experiences and improved pain communication with their providers [24]. The other two studies indicated total number of reports completed (9216 entries [25] and >13,000 assessments and 4329 person days [26]) without any description of participant acceptability. See Multimedia Appendix 2 for usage and acceptability outcomes.

### Intervention

#### Study Characteristics

Eleven studies reported interventions targeting mood, anxiety, or wellness outcomes for pediatric populations with socially complex needs. Specifically, interventions targeted (1) sexual risk behaviors in (i) African American adolescent females [27], (ii) at-risk adolescent females from a remote area in West Africa (Ghana) [32], and (iii) sexual minority adolescent males [37]; (2) obesity-related behaviors in (i) racial/ethnic minority adolescent females from low SES homes [29], (ii) adolescent males from low SES homes [35], and (iii) adolescents from diverse racial/ethnic backgrounds [36]; (3) asthma adherence in racial/ethnic minority adolescents from low SES homes [30,34]; (4) emotion regulation in homeless adolescent mothers [28]; (5) emotional self-awareness and mood symptoms in adolescents and young adults treated in rural primary care settings [31]; and (6) pain coping in racial/ethnic minority adolescents with SCD [33]. All samples were American, with the exception of 2 from Australia [31,35] and 1 from Ghana [32]. Samples ranged in size from 26 to 701, and nearly half consisted entirely of one sex [28,30–32,36,37]. See Table 1 for study characteristics.

#### Population-Specific Tailoring

Four studies explicitly described tailoring of the intervention to the targeted population. DiClemente and colleagues [27] utilized health educators matched by race and sex to the participants for the in-person session (ie, African American females) and described specifically tailoring the telephone counseling strategies to address sexual risk factors as prioritized by the participants (eg, a partner declining to wear a condom). Using participants themselves to tailor the intervention, Seid and colleagues [34] reported that participants created their own messages that would be sent as SMS text message during the intervention period. Examples included behavioral cues (eg, “Take your meds and go exercise.”), motivational messages based on personal reasons for change (eg, “Don’t quit. You can do it.”), and general queries (eg, “Doing okay with your asthma? If not, call…”). Thompson et al [36] depicted both in text and through reference of an earlier work [39] an iterative approach to developing 84 SMS text message prompts (12 goal prompts, 72 promoting psychological needs; equally grounded in autonomy, competence, and relatedness) with 160 adolescents who were primarily racial/ethnic minorities. Examples included SMS text messages grounded in autonomy (eg, “You’re in charge! Make the choice to meet your step goal today!”), competence (eg, “You can meet your step goal; just keep steppin’!”), and relatedness (eg, “Meeting your step goal shows you have what it takes to be successful!) [36]. Finally, Ybarra and colleagues [37] tailored messages based on sexual experience. For example, sexually experienced teens received an SMS text message such as “When you’re in a healthy relationship and start having sex…,” whereas sexually inexperienced teens would view: “When you have sex…” [37].

Three studies cited previous work depicting iterative design input from potential end users. While Leonard et al [28] reported qualitative feedback from their participants about their system, they cited a prior pilot conducted with 4 African American or Latinx adolescent mothers from low SES families at high risk for school dropout. Findings indicated participants’ desire to make the sensor bands that measured electrodermal activity (a physiological stress indicator) more comfortable and fashionable in appearance [40]. Nollen et al [29] cited previous work that formed a community advisory board (CAB) of adolescent girls, who were primarily racial/ethnic minorities, to provide feedback on the use of the technology platform and to test 2 iterations of prototypes of the intervention. The CAB requested more reminders, accountability monitoring, and free music as an incentive for use; these were incorporated in later iterations of the design [41]. Reid and colleagues [31] cited previous focus groups with high-school students to tailor their app question prompt language (eg, make it less repetitive) [42].

Four studies provided vague or no descriptions of tailoring the intervention to a specific population. While Perry et al [30] provided limited detail on tailoring (eg, colorful graphics), they described seeking input from community stakeholders (teens with asthma and their parents). Rokicki and Fink [32] reported integrating feedback from adolescent focus groups and health providers to design the intervention language; however, no examples were provided. Smith and colleagues [35] described using tailored informational and motivational SMS text messages that were pushed to participants without providing details of this tailoring. Finally, to the best of our knowledge, Schatz and colleagues [33] did not describe tailoring in any way.

#### Evidence-Based Skills and Theories

The included studies reported the use of evidence-based skills and grounding in multiple theories. The most common evidence-based treatments were cognitive behavioral therapy (CBT) [28,33], skills related to CBT (eg, behavior modification through goal setting and self-monitoring) [29,41], mindfulness [28], problem-solving skills training [34], and motivational interviewing [34]. Disease-specific interventions and models of change were also noted for asthma (asthma action plans) [30] and HIV (Information-Motivation-Behavior Model of HIV) [37]. Finally, self-determination theory [35,36], social cognitive theory [27,35], and the theory of gender and power [27] were used to inform several interventions. These theories were not necessarily used in isolation. For example, DiClemente and colleagues [27] grounded their intervention (HORIZONS) in both evidence-based treatment [43,44] and minority-based theory [45,46].

#### Use of Phones for Delivery

Consistent with the inclusion criteria, all studies used phones for some means of intervention delivery. Included in 5 studies, smartphone apps were the most commonly reported phone-based delivery mechanism [29-31,33,35], followed by the use of SMS text messaging, in 4 studies [32,34,36,37]. A total of 5 studies described providing some or all of their participants with a mobile phone or smartphone for the duration of the study [31,35–38]; 1 study did not clarify whether participants used their own devices [35]. Two studies required that participants have their own mobile phone with unlimited SMS text message/data plans [36,37]. Finally, DiClemente and colleagues [27] described the delivery of brief, tailored telephone-delivered counselling sessions following a single, in-person training session.

#### Additional Support

With one exception [29], all studies included some form of additional support to participants. Five studies included at least one in-person therapy or skills training session [27,28,33-35], with one additional study including training for teachers who would be interacting with participants throughout the intervention [35]. Four studies utilized automated support in the form of SMS text messages or reminders [29,34–36]. Three studies provided remote support with telephone-delivered counseling or check-ins [27,33] or being paired with a “text buddy”—another participant matched on sexual experience (ie, experienced or inexperienced) within 1 time zone but at least 500 mi away [37]. Finally, 3 studies reported using “as needed” remote human support via telephone calls [31,33] or SMS text messaging [36], which was activated when there were concerns for safety or poor adherence.

#### Primary Outcomes

The primary outcome measures and results varied considerably across the 11 intervention studies. Three studies reported using intent-to-treat analyses [27,31,37]; 2 studies reported nonsignificant primary outcomes [36,37] and 2 reported changes in knowledge or behaviors without noting significance values [28,32]. For pediatric condition-specific interventions, those with (1) uncontrolled asthma had improved asthma control test scores following use of the asthma action plan app (*P*=.04) [30]; (2) asthma that had received tailored SMS text messages had medium to large effect size changes in asthma symptoms and health-related quality of life [34]; and (3) SCD demonstrated a group (CBT training and app vs waitlist control) × time interaction for coping attempts (*P*=.03) [33]. For obesity-related behavior interventions reporting significant findings, the use of an app targeting obesity-related behaviors in racial/ethnic minority adolescent females from low SES homes was associated with less sweetened beverage consumption (*P*=.01) [29], whereas use of an app with a school-based program was related to changes in screen time (*P*=.03), lowered sweetened beverage consumption (*P*=.01), increased muscular fitness (*P*=.04), and increased resistance training skills (*P*=.001) [35]. To target sexual risk behaviors in adolescent females, those receiving telephone counseling were less likely to have a chlamydial infection (*P*=.02) or report having sex while high (*P*<.001), and more likely to use a condom (*P*=.04) [27]. Finally, in an app targeting emotional self-awareness and mood symptoms in adolescents and young adults treated in rural primary care settings, there was a group (monitoring mood symptoms vs daily activity monitoring) × time interaction effect for emotional self-awareness (*P*=.048) and main effects for depression and anxiety symptoms (*P*<.02 for both) [31]. See Multimedia Appendix 2 for primary outcomes.

#### Usage and Acceptability

One study did not report usage data [34] and 5 did not report acceptability [28–30,37,38]. Usage was reported in variable ways, including percentage of sample that used or reported using the SMS text messages/app [32,35,36], percentage of completed entries [33], average daily SMS text messages [37], total number of calls [27], duration of app use [28], percentage of days used [29], and frequency (eg, days per week or times per week) [30]. Three studies reported a numeric rating for acceptability or satisfaction with the intervention [28,29,36]. Two studies provided the percentages of their sample who agreed with statements such as “I would recommend the app to a friend with asthma” [30,35]; one study described participants as finding the intervention to be “appealing” [34]. See Multimedia Appendix 2 for usage and acceptability outcomes.

### Quality of Studies

The included studies ranged from tracking and assessment to interventions, indicating that different methodologies were anticipated. Indeed, the 3 tracking and assessment studies, by the very nature of their purpose, were deemed high risk for selection, performance, and detection biases (Multimedia Appendix 3) [23]. Further, allocation concealment as well as performance and detection biases were variable, likely due to the nature of frequently involving technological delivery mechanisms (eg, knowing which arm a participant is assigned because they have access to an app or not). Attrition bias was high for 1 tracking and assessment study [24], but low (11/14, 79%) or unclear (2/14, 14%) for all other studies. Finally, all studies had a low reporting bias.

## Discussion

### Principal Findings

This study synthesized the literature on the use of phones (ie, mHealth and telehealth) as a mental and behavioral health delivery mechanism for pediatric populations with socially complex needs. There was high variability in methodological approaches and reporting of data, negating the possibility of a meta-analytic approach to this systematic review. The studies that met the inclusion criteria were primarily intervention based and occurred mainly in 2005-2007 (we assumed that these studies primarily used landline calls) [27]. Samples included targeting typically underserved populations by gender [28–32,36], racial/ethnic background [24,25,28,33–35], low SES status [26,31,32,34–36], rural/remote location [31,32], and sexual orientation [37]. Usage and acceptability of the delivery mechanisms were inconsistently reported and therefore difficult to generalize. Finally, given the nature of the included studies, risk of bias to issues such as blinding was generally high.

The purpose of this synthesis of the literature was to provide recommendations for future research developing phone-based interventions for youth with socially complex needs. The following sections will therefore be used to discuss implications of the current findings for the development of future interventions targeting such pediatric groups. Specifically, we discuss (1) uses of phone-based delivery practices, (2) culturally specific tailoring practices, (3) applications of evidence-based skills and grounded theories to inform design, and (4) additional supports that promote comfort, use, or intended intervention outcomes for pediatric populations with socially complex needs.

#### Phone-Based Delivery

Given the ubiquity of mobile phones and smartphones [14], the use of these devices to reach socially complex pediatric populations has great merit. This focus on mobile devices stands in contrast to previous efforts to deliver evidence-based treatments via computer-based platforms [47,48] and may more accurately reflect the device and broadband access of underserved communities [12]. Further, phones are now equipped with multiple access capabilities, such as the ability to provide context sensing and just-in-time interventions (ie, acting when youth are most likely to be in need of in-the-moment intervention) [49,50]. It is possible that more interventions for socially complex pediatric populations will be entirely encompassed within mobile platforms, including multimethod (eg, context sensing, calls, SMS text messages, and an app), just-in-time, or stepped care designs (eg, early nonresponders step up care from SMS text messaging only to SMS text messaging + app, to SMS text messaging + app + telephone-administered CBT).

Apps and SMS text messaging were the most commonly employed method to access youth. This is consistent with current usage trends, such that youth are more likely to use SMS text messaging or social media to communicate than phone calls [51]. While apps are numerous and widely accessible, adoption is often poor [52] and there appear to be gaps in coverage across development (eg, apps aimed primarily at children or adults, but fewer for teens). Further, pediatric clinical-scientists are unlikely to develop, evaluate, and disseminate apps in pace with industry-driven apps [53], making the development of future apps targeted specifically for unique pediatric conditions or samples less feasible without industry support. Therefore, despite apps and SMS text messaging both being the most frequently used within the studies included in this review, we venture that there may be benefit in also focusing on using SMS text messages to assess and intervene with pediatric samples with socially complex needs. Indeed, SMS text messaging interventions are (1) low cost (for interventionists; please see below about discussion of potential costs for users); (2) consistent with technology practices identified within several underserved population groups [14]; (3) not as easily ignored as push notifications and do not require a user to open a specific or potentially “identifiable” app (eg, a teen might fear that a specific app would be recognized by a peer for treating depression); (4) and associated with improvements in behavioral health behaviors for general pediatric and pediatric populations from underserved communities [16,54–56]. While the future of phone-based delivery of pediatric interventions may be multifaceted, apps and SMS text messaging appeared frequently in the current literature. We posit that SMS text messaging may be a particularly viable option for engaging populations with socially complex needs in pediatric assessments and interventions.

Relevant to the use of phones, potential costs associated with the use of phones as mental and behavioral health delivery mechanisms are also worth noting. Indeed, this consideration is particularly crucial in trying to access certain socially complex populations who are more likely to be impacted by lapses in service or burdened by the cost of data packages/SMS text messaging plans [15,18]. Several studies provided phones or data/SMS text messaging plans to participants, whereas others required that participants already own a smartphone with unlimited data/SMS text messaging plans. Previous work has already described design recommendations to avoid hidden costs to users (eg, data downloads when connected to Wi-Fi) [54]. However, ongoing assessments relating to the costs or burdens of mHealth, telehealth, and other use of future digital mental health tools should be conducted with representative pediatric populations and their families.

#### Culturally Specific Tailoring

Informing design with the feedback and preferences of likely end users is an integral aspect of user-centered design practices [55]. Cultural tailoring should therefore not be a unique practice. However, as there is limited literature targeting pediatric patients with socially complex needs (ie, only 14 studies meeting inclusion criteria for this review), such tailoring has rare representation. Involvement of representative end users in the design process was described in varying detail across studies. Given the paucity of direction for designing for specialty populations, papers specific to development (eg, [40–42]) or more explicit depictions of culturally specific tailoring are critically necessary for future publications.

Involving representative end users (eg, pediatric populations with socially complex needs) in design decisions may be achieved through multiple means. For example, the current literature detailed the use of semistructured individual interviews, focus groups, membership in a CAB, and stakeholder involvement. Recruiting youth to participate in such activities likely requires multimethod strategies, including flyers or targeted electronic chart messaging from pediatric primary care, school-based health centers, or specialty clinics. Engaging community organizations in partnership to engage youth will also increase the likelihood of receiving input from populations who have been historically less involved in research. Examples from the current literature depicted recruitment through specialty clinics (eg, SCD treatment site) or community settings to aid in tailoring messages or determining workflow of the interventions. Message tailoring was achieved through engaging representative groups, using messages authored by individuals for themselves [34], or by altering language based on membership to a given category (eg, sexually experienced vs inexperienced) [37]. As noted above, the authors posit that SMS text messaging stands as a viable means to engage underserved pediatric populations in assessment and interventions. Language utilized in such messages must be appropriate for the youth’s needs, requiring brevity, clarity, minimal jargon, inclusive language choices, and the avoidance of a condescending tone—particularly for adolescent users [20,56,57]. It seems unlikely that such goals for language could be achieved without the direct input of the populations who would be using the tools. Indeed, as intervention design is inherently led by adults, the authors venture that beyond user-centered design practices, all pediatric interventions should have some form of input from youth. Regarding workflow, flexibility appears to be critical. Indeed, welcoming input from potential users about what they view as their top priorities and needs likely promotes engagement and usability.

#### Applications of Evidence-Based Skills and Grounded Theories

Also integral to the methodologies of interventions targeting pediatric populations with socially complex needs is grounding the design in evidence-based treatments. Skills grounded in CBT were most commonly employed to achieve the intended behavioral change in the studies meeting inclusion criteria. However, theories relating to minority populations (eg, promoting power, equity) were also used to guide the interventions. As members of underserved populations tend to have intersectional identities (eg, individual identification with minority status in sex/gender [female], ethnicity [Latinx], and SES [low SES]), grounding in theory likely also requires an intersectional approach. For example, in targeting sexual risk behaviors in African American adolescent females, the HORIZONS intervention was grounded in multiple theories, incorporating both an evidence-based treatment [43,44] and a minority-based theory [45,46]. Future interventions for underserved pediatric populations will likely benefit from similar integrative models of theory.

#### Additional Supports

Additional supports, whether automated, human, or both, also appear to be important for interventions targeting pediatric populations with socially complex needs. The potential for automated responses promotes the scalability of interventions for specific population targets. However, relating to the specialty needs of varying pediatric populations who may also be from underrepresented groups, the ability to have support as needed or ongoing human support may also be important. Fortunately, the incorporation of human support further opens up the possibility of increased diversity in the workforce that designs and deploys such interventions. Indeed, the US Department of Health and Human Services’ Office of Minority Health argues that one way to improve health disparities is by increasing clinical workforce diversity [58]. Ideally, human support staff (eg, health educators, clinicians, researchers) should therefore include demographic membership that is in some way representative of the patients being served. For example, DiClemente and colleagues [27] ensured that all in-person contacts (ie, recruitment, health education sessions) were staffed by professionals matched by gender (female) and race (African American) to the participants [27]. Such inclusive hiring and collaborative processes likely enhance patient engagement and further promote cultural-tailoring practices highlighted above.

### Limitations

This systematic review should be interpreted in light of specific limitations. First, the studies meeting inclusion criteria were incredibly variable in their methodologies and reporting strategies. This variability precluded a meta-analytic data approach to data synthesis and we were hesitant to overinterpret outcomes and usage patterns. Related to the variability in the studies, the search for research about “pediatric populations with socially complex needs” comprises a broad group. The current findings should be interpreted in terms of broad application to these pediatric populations. Second, the inclusion criteria for this systematic review led to the exclusion of more broad applications of mHealth and telehealth interventions for pediatric populations (eg, samples that included primarily majority population participants). It is unclear how larger reviews of the literature for pediatric populations may generalize to the populations targeted in this review, and vice versa [59]. Third, the included studies were conducted in the context of specific research trials. It is unclear how the findings generalize to open deployment and if there are specialty concerns for specific underserved groups (eg, regional differences). Further, a number of studies did not report postintervention follow-up data. It is therefore difficult to identify potential long-term impacts of the interventions. Finally, and as previously noted, we were also limited in our ability to synthesize cultural-tailoring practices, as multiple included studies did not explicitly report these methodologies. Indeed, future detailed depictions of design practices identified with and for specific underserved pediatric populations are needed going forward—in both primary outcome reports and reviews.

### Conclusions

Repeated calls have been made to better target the behavioral health needs of pediatric patients with socially complex needs. Mobile devices are often owned and utilized, and therefore may be an optimal delivery mechanism to access youth from such groups. Given the need to focus such interventions to the specialized needs of socially complex youth, this study systematically reviewed the literature of phone-based interventions (mHealth and telehealth) aimed at mental and behavioral health targets for pediatric populations. The synthesis highlighted the importance and potential opportunities of (1) the involvement of representative end users, stakeholders, and clinical/research staff; (2) integration of evidence-based therapies with minority-focused theories; (3) harnessing the capabilities of mobile devices, including SMS text messaging; and (4) considering and assessing for potential costs related to phones as delivery mechanisms. Future research should promote such practices and explicitly detail population-specific tailoring, usage, and acceptability of interventions delivered via mobile devices.

## Appendix

Multimedia Appendix 1 Search strategy and terms.

Multimedia Appendix 2 Study outcomes.

Multimedia Appendix 3 Study risk of bias.

## Abbreviations

CAB: community advisory board
CBT: cognitive behavioral therapy
EMA: ecological momentary assessment
mHealth: mobile health
PRISMA: Preferred Reporting Items for Systematic Reviews and Meta-Analyses
SCD: sickle cell disease
SES: socioeconomic status
