# Supplementary material for: Harnessing Phones to Target Pediatric Populations with Socially Complex Needs: Systematic Review
Source: JMIR Pediatr Parent. 2020 Aug 26;3(2):e19269. doi: 10.2196/19269 (PMC7481873; doi:10.2196/19269)
Supplement: Multimedia Appendix 1 [file pediatrics_v3i2e19269_app1.docx]

| **Telehealth** | **Marginalized** | **age** |
| --- | --- | --- |
| eHealth  or mHealth  or landline or landlines  or phone or phones  or smartphone or smartphones  or "digital mental health"  or internet-based  or internet-delivered  or telehealth  or telemedicine  or Telepsychiatry  or telepsychology  or teleneurology  or tele-intensive  or telepediatric  or telemental  or telemonitoring  or telepractice  or “remote monitoring”  or videoconference  or videoconferencing  or texting  or app or apps  or “text messaging”  or “text message” or “text  messages”  NOT sexting  OR ((phone or phones  or smartphone  or smartphones)  AND (application  or applications  or mobile  or (text not “full text”) or texts)) | disparity or disparities  or ethnic*  or indigenous  or marginalized  or minority or minorities  or poverty  or remote  or rural  or segregation  or socioeconomic  or underserved  or vulnerable  or pain  or disabled  or deaf  or “hearing loss”  or blind or “vision impaired”  or handicapped  or haemophilia or hemophilia or discrimination | adolescen*  or boy or boys  or (child* NOT “child abuse”)  or girl or girls  or juvenile*  or kid or kids  or minors or minors  or paediatric or paediatrics  or pediatric or pediatrics  or prepubescent  or pre-pubescent  or pubescent  or PICU  or schoolchild*  or teen or teens  or teenager or teenagers  or toddler* or youth* |
| NOT (protocol Or survey* or questionnaire* or dental or periodontal or “Internet addiction” or “Maternal Health”) | | |
