# Supplementary material for: Harnessing Phones to Target Pediatric Populations with Socially Complex Needs: Systematic Review
Source: JMIR Pediatr Parent. 2020 Aug 26;3(2):e19269. doi: 10.2196/19269 (PMC7481873; doi:10.2196/19269)
Supplement: Multimedia Appendix 2 [file pediatrics_v3i2e19269_app2.docx]

Supplementary Table 1.

*Study Outcomes*

| Authors, Year | Primary Measures | Primary Outcome | Usage Outcome | User Acceptability |
| --- | --- | --- | --- | --- |
| Tracking and Assessment | | | | |
| Bakshi et al., 2017 | Pain Intensity Score | 30% with all reports having pain (median = 178.5; 16-622 pain reports); 30% without pain most of time (median = 79.5; 19-392 no pain reports) | >85% of reported days associated with 2+ reports | Focus groups report generally positive experience |
| Jacob et al., 2013 | Symptoms Checklist; VAS, Adolescent Pediatric Pain Tool | 55.2% of entries reported pain; *M* Pain = 4.1 ± 2.2 (range: 1-10) | 9216 diary entries | NR |
| Odgers et al., 2017 | Self-report of violence exposure; Modified/adapted BDI, PANAS, ARI, CBCL, Youth Risk Surveillance Survey | 75% exposed to violence ≥1 study days; ≥1 depressive symptom on 25.9% of days, Anger/Irritability on 14.6% of days, Conduct problem symptoms on 7.7% of days, Health-risk behavior on 13.1% of days | >13,000 assessments and 4,329 person days obtained | NR |
| Intervention | | | | |
| DiClemente et al., 2014 | Lab-confirmed STI; Self-report of unprotected sex and/or sex while under the influence of a substance | Experimental condition: less likely to have a chlamydial infection (*p* = .02), report having sex while high (*p* <. 001) and more likely to use a condom (*p* = .04); Sample: less likely to have a chlamydial infection with more telephone contact (*p* = .05) ^a^ | *M* Calls: 10.78 ± 5.44 in the experimental condition, 9.86 ± 5.22 in comparison condition (*p* = .02). | NR |
| Leonard et al., 2018 | Electordermal activity; Self-reported emotion regulation | Self-reported valence was negative (*M* = 47.30 +/- 16.50); Sensorband-initiated reports were positive (57.23 +/- 11.2) | Phone Access: 155.8 ± 75.16 (15-316 days); App Use: 14.77 ± 14.39 (605.67 minutes) | Acceptability 3.55/4; 75% “Very Satisfied” |
| Nollen et al., 2013 | 24-hour dietary recall; Brief Questionnaire of Television Viewing and Computer Use; BMI | Use associated with less sweetened beverage consumption (*p* = 0.01); All other outcomes NS | Used 63% of days of the study | Average enjoyment 4.5 ± 0.9 |
| Perry et al., 2016 | ACT; Child Self-Efficacy Questionnaire | Uncontrolled asthma group improved ACT scores (*p* = .04); All other outcomes NS | Median of 4.36 days/week and 12.17 times/week | 92%: like to continue using the app; 100%: would recommend the app to a friend with asthma |
| Reid et al., 2011 | DASS; ESA | Group × time interaction for ESA (*p* = .048); Main effect for time for depression (*p* < .001), anxiety (*p* = .02) ^a^ | Daily entries: 3.3 ± 1.4 (1-8/day); 52.9% participants in intervention and 60.9% in comparison received a minimum dose | NR |
| Rokicki et al., 2017 | Health Knowledge Quiz | Increase in SRH knowledge in all domains; Engagement associated with higher knowledge scores at follow-up | 81% of sample engaged with platform | NR |
| Schatz et al., 2015 | Coping Strategies Questionnaire for SCD; Electronic Daily Pain and Activity Diary | Group × time interaction for coping attempts (*p* = .032) | 61% Diaries complete (1547/2530) | NR |
| Seid et al., 2011 | PedsQL Asthma Module Treatment Problems Scale and Generic Core Scales | Improvements in asthma symptoms (Cohen’s *d*s 0.40, 0.96) and QOL (Cohen’s *d*s 0.23, 1.25) | NR | Participants found the intervention to be “appealing” |
| Smith et al., 2014 | BMI, waist circumference, % body fat, Accelerometer output, screen-time, sugar-sweetened beverage intake, muscular fitness, resistance training skill competency | Changes in screen-time (*p* = .03), sugar-sweetened beverage consumption (*p* = .01), muscular fitness (*p* = .04), resistance training skills (*p* = .001) | 63% self-reported using the app | 44% Agreed/Strongly agreed use was "enjoyable" |
| Thompson et al., 2016 | Step count, moderate-to-vigorous activity | NS | 85% self-reported reading messages daily | *M* satisfaction = 17.47/20 |
| Ybarra et al., 2017 | CSAs, Abstinence 90 days posttreatment | NS ^a^ | Mean daily texts: 8.5 (Inexperienced), 9.6 (Experienced), 7 (Control) | NR |

*Note*. ACT = asthma control test; ARI = Affective Reactivity Index; BDI = Beck Depression Inventory; BMI = body mass index; CBCL = Child Behavior Checklist; CSA = condomless sex acts; DASS = Depression, Anxiety, Stress Scale; ESA = Emotional Self-Awareness Scale; M = mean; NR = not reported; NS = not significant; PANAS = Positive and Negative Affect Schedule; QOL = quality of life; SCD = sickle cell disease; SRH = sexual and reproductive health; STI = sexually transmitted infection; VAS = Visual Analog Scales.

^a^ Based on intent-to-treat (ITT) analyses
