# Supplementary material for: Harnessing Phones to Target Pediatric Populations with Socially Complex Needs: Systematic Review
Source: JMIR Pediatr Parent. 2020 Aug 26;3(2):e19269. doi: 10.2196/19269 (PMC7481873; doi:10.2196/19269)
Supplement: Multimedia Appendix 3 [file pediatrics_v3i2e19269_app3.docx]

Supplementary Table 2.

*Study Risk of Bias*

| Authors, Year | Selection Bias: Random Sequence | Selection Bias: Allocation Concealment | Performance Bias | Detection Bias | Attrition Bias | Reporting Bias |
| --- | --- | --- | --- | --- | --- | --- |
| Tracking and Assessment | | | | | | |
| Bakshi et al., 2017 | High | High | High | High | High | Low |
| Jacob et al., 2013 | High | High | High | High | Low | Low |
| Odgers et al., 2017 | High | High | High | High | Low | Low |
| Intervention | | | | | | |
| DiClemente et al., 2014 | Low | Low | Medium | Low | Low | Low |
| Leonard et al., 2018 | High | High | High | High | Low | Low |
| Nollen et al., 2013 | Low | Unclear | High | Unclear | Low | Low |
| Perry et al., 2016 | Low | High | High | High | Low | Low |
| Reid et al., 2011 | Low | Low | Medium | Low | Low | Low |
| Rokicki et al., 2017 | Low | High | High | Unclear | Unclear | Low |
| Schatz et al., 2015 | Low | Low | Medium | Low | Unclear | Low |
| Seid et al., 2011 | Low | Low | Medium | High | Low | Low |
| Smith et al., 2014 | Low | Low | High | High | Low | Low |
| Thompson et al., 2016 | Low | Low | Unclear | Unclear | Low | Low |
| Ybarra et al., 2017 | Low | Low | Unclear | Low | Low | Low |
